# Upregulation of Anti-Angiogenic miR-106b-3p Correlates Negatively with IGF-1 and Vascular Health Parameters in a Model of Subclinical Cardiovascular Disease: Study with Metformin Therapy

**DOI:** 10.3390/biomedicines12010171

**Published:** 2024-01-12

**Authors:** Sherin Bakhashab, Josie O’Neill, Rosie Barber, Catherine Arden, Jolanta U. Weaver

**Affiliations:** 1 Biochemistry Department, King Abdulaziz University, P.O. Box 80218, Jeddah 21589, Saudi Arabia; sbakhashab@kau.edu.sa; 2 Translational and Clinical Research Institute, Newcastle University, Newcastle upon Tyne NE2 4HH, UK; josieoneill@virginmedia.com (J.O.); rosie_barber@icloud.com (R.B.); 3 Center of Excellence in Genomic Medicine Research, King Abdulaziz University, P.O. Box 80216, Jeddah 21589, Saudi Arabia; 4 Biosciences Institute, Newcastle University, Newcastle upon Tyne NE2 4HH, UK; catherine.arden@newcastle.ac.uk; 5 Department of Diabetes, Queen Elizabeth Hospital, Newcastle upon Tyne NE9 6SH, UK; 6 Vascular Biology and Medicine Theme, Newcastle University, Newcastle upon Tyne NE1 7RU, UK

**Keywords:** IGF-1, IGFBP-3, miR-106b-3p, CFU-Hill colonies, subclinical CVD, metformin

## Abstract

Well-controlled type 1 diabetes mellitus (T1DM) is regarded as a model of subclinical cardiovascular disease (CVD), characterized by inflammation and adverse vascular health. However, the underlying mechanisms are not fully understood. We investigated insulin-like growth factor-1 (IGF-1) and IGF-binding protein-3 (IGFBP-3) levels, their correlation to miR-106b-3p expression in a subclinical CVD model, and the cardioprotective effect of metformin. A total of 20 controls and 29 well-controlled T1DM subjects were studied. Plasma IGF-1, IGFBP-3 levels, and miR-106b-3p expression in colony-forming unit-Hills were analyzed and compared with vascular markers. miR-106b-3p was upregulated in T1DM (*p* < 0.05) and negatively correlated with pro-angiogenic markers CD34+/100-lymphocytes (*p* < 0.05) and IGF-1 (*p* < 0.05). IGF-1 was downregulated in T1DM (*p* < 0.01), which was associated with increased inflammatory markers TNF-α, CRP, and IL-10 and reduced CD34+/100-lymphocytes. IGFBP-3 had no significant results. Metformin had no effect on IGF-1 but significantly reduced miR-106b-3p (*p* < 0.0001). An Ingenuity Pathway analysis predicted miR-106b-3p to inhibit PDGFA, PIK3CG, GDNF, and ADAMTS13, which activated CVD. Metformin was predicted to be cardioprotective by inhibiting miR-106b-3p. In conclusion: Subclinical CVD is characterized by a cardio-adverse profile of low IGF-1 and upregulated miR-106b-3p. We demonstrated that the cardioprotective effect of metformin may be via downregulation of upregulated miR-106b-3p and its effect on downstream targets.

## 1. Introduction

Cardiovascular disease (CVD) is the primary cause of death worldwide, encompassing approximately a third of total annual deaths [1] with an estimated 18 million people [2]. Various comorbidities have been shown to increase CVD risk, one being diabetes. CVD complications that arise from having T1DM for a prolonged period have been seen to significantly shorten a person’s lifespan by more than 10 years [3]. The risk of CVD mortality is still increased by almost three times in those with T1DM even when it is well-controlled with stable glycemic levels [4].

Previous case-control work has shown that well-controlled T1DM can be regarded as subclinical CVD, owing to endothelial dysfunction, increased inflammatory markers and circulating endothelial cells (cECs), and decreased levels of circulating endothelial progenitor cells (cEPCs), colony-forming unit-Hills (CFU-Hills), and pro-angiogenic cells (PACs) [5,6,7,8,9,10]. Subclinical CVD is an asymptomatic early indicator of future clinical CVD. This, therefore, makes well-controlled T1DM a good model to study, as understanding the development of CVD can aid in developing future therapies to potentially prevent progression to clinical CVD.

ECs and EPCs are commonly used as biomarkers to evaluate future CVD risk. High levels of cECs imply that the endothelium has become injured, causing ECs to become dislodged and enter circulation [11]. cECs are therefore used as biomarkers of endothelial damage, with elevated levels observed in many cardiovascular disorders and being predictive of poor outcomes [12,13,14]. EPCs circulate in the blood and find sites of vascular or endothelial damage to aid their repair by promoting neovascularization through secretion of growth factors such as vascular endothelial growth factor (VEGF) and insulin-like growth factor 1 (IGF-1) [15] and differentiate into mature EPCs, which attach to injured endothelium [16,17]. cEPCs are therefore used as biomarkers of endothelial function, with reduced levels seen in cardiovascular conditions [7,18,19,20]. Colony-forming unit-Hills (CFU-Hills) are a form of early EPC [7,21,22] comprised of various cell types such as monocytes, leukocytes, and EPCs [23]. They have a low proliferative capacity and indirectly influence angiogenesis via secreting paracrine factors such as growth factors and chemokines [22]. CFU-Hills are important for angiogenesis and have been shown to be inversely correlated to the Framingham risk score for cardiovascular health [7,23]. Low levels have been connected to endothelial dysfunction and CVD risk factors [7,24].

Insulin-like growth factor 1 (IGF-1) is one of the growth factors secreted by EPCs and has an important role in the cardiovascular system. IGF-1 is a small peptide in the IGF family of insulin-related peptides produced primarily by the liver. It has pleiotropic effects, many of which are implicated in maintaining cardiovascular health [25,26]. The availability of free IGF-1 in circulation is modified by different IGF-binding proteins (IGFBPs) which form complexes with it and regulate its effects. The biological half-life of IGF-1 in circulation is lengthened by forming complexes with IGFBPs, which generate a pool of easily accessible IGF-1 to then be distributed to different tissues. The most plentiful IGFBP in adult serum is IGFBP3, which is the major circulating carrier of IGF-1 [27]. Low levels of IGF-1 and IGFBPs have been linked to diabetes and CVD risk [28]. This is due to the role of IGF-1 in the regulation of insulin metabolism as well as proliferation, differentiation, and apoptosis in a variety of cell types [27,29]. However, the role of IGF-1 in relation to EPCs in subclinical CVD has not yet been fully studied. Therefore, there is a need for more research into IGF-1 and IGF-1-associated pathways in CVD development in subclinical CVD models such as T1DM.

Emerging studies suggest the use of miRNAs as their dysregulation is thought to play an important part in the pathogenesis of diseases such as T1DM and CVD [30,31]. Research has associated miR-106b with skeletal muscle insulin resistance [32], which is often seen in T1DM. However, there is a discrepancy in the role of miR-106b-3p in CVD. In rat models, miR-106b-3p was found to be downregulated in congestive heart failure [33]. Whereas another study discovered that upregulated miR-106b-3p was associated with atrial fibrillation in patients [34]. High miR-106b levels were also detected in microparticles released from atherosclerotic plaques [35]. In retinal mouse models, reducing miR-106b levels initiates pro-angiogenic protein production, prompting vascular growth and neovascularization [36].

Twenty-six years ago, metformin was identified to have cardioprotective properties in T2DM [37]. In further studies, in patients with T2DM, metformin monotherapies have shown the reduction of all-cause mortalities and CVD events [38]. In addition, metformin has been shown to have various actions that reduce CVD or CVD risk beyond modification of glycemic control: 1. By lowering vascular inflammation [39], 2. By improving hypertension, having antioxidant effects [40], and 3. By improving endothelial function [41,42]. Animal models have demonstrated metformin’s cardio-protection by preserving cardiac function post-MI or reperfusion injury, or by promoting angiogenesis and neurogenesis in mice following middle cerebral artery occlusion [43,44,45]. In the REMOVAL trial in patients with T1DM, metformin has been shown to significantly reduce maximal carotid artery intima-media thickness (cIMT) [46]. In a parallel study, but with unchanged glycemic control (MERIT study), metformin’s benefits in T1DM patients included improvement of cEC and cEPC levels [5]. Some other possible cardioprotective mechanisms of metformin may be through altering the IGF axis [27], or through its role in regulating pathways associated with cardio-adverse miRNAs [42,47]. However, this is not conclusive, and so further studies are required.

We hypothesized that: (1) IGF-1 and IGFBP-3 levels are decreased in T1DM and elevated by metformin, and (2) miR-106b-3p expression in CFU-Hills is upregulated in T1DM while downregulated by metformin. We aimed in this study to explore the role of miR-106b-3p as a subclinical CVD biomarker and the effect of metformin on miR-106b-3p expression in T1DM patients.

## 2. Materials and Methods

### 2.1. Participants

A total of 29 T1DM patients and 20 age- and gender-matched healthy controls (HCs) were recruited into the MERIT study. T1DM patients’ inclusion criteria included HbA1c <8.5% (69mmol/mmol) and no presence of diabetic complications such as evident CVD. T1DM participants were administered metformin for 8 weeks to the highest tolerated dose or with a dose titrated up to a maximum of 1 g twice a day [5]. The sample size for the MERIT study was calculated and has been previously published [5]. The minimum number of subjects required in each group was 20.

Approval for this study was given by the NHS Health Research Authority, NRES Committee Northeast-Sunderland, UK (Research Ethics Committee Reference Number 12/NE/0044) and completed in keeping with the Helsinki Declaration. Written informed consent was obtained from all participants in this study before its commencement.

### 2.2. Meso Scale Discovery (MSD) Assay

Cytokine levels investigated in this study were measured in the MERIT study from subject plasma samples and assayed using human Meso Scale Discovery panels (Meso Scale Discovery, Rockville, MD, USA), as previously detailed [8].

### 2.3. Flow Cytometric Evaluation of cECs and cEPCs

Analysis of cECs and cEPCs was performed using flow cytometry on a BD FACS CantoTM II system (BD Bioscience, San Jose, CA, USA) as previously detailed [5]. cEPCs were identified as CD45dimCD34+VEGFR-2+ cells and cECs as CD45dimCD133−CD34+CD144+. Proangiogenic cell (PAC) in vitro assay and fibronectin adhesion assay (FAA) were previously described by us [5].

### 2.4. Culture of CFU-Hill Colonies

CFU-Hill colonies were cultured according to the method described previously [8].

### 2.5. Real-Time Quantitative PCR and miRNA Expression

Total RNA was isolated using the miRNeasy Micro Kit (QIAGEN, Hilden, Germany), as explained in Phowira et al., 2022 [8].

Reverse transcription was performed using the miRCURY LNA RT Kit (QIAGEN, Hilden, Germany) on about 10 μL of RNA. Following the methodology for miRCURY LNA miRNA PCR, cDNA was diluted 100× and assessed in 10 μL PCR reactions. The miRNA was assayed once by qPCR using the miRCURY LNA SYBR Green master mix on the miRNA Ready-to-Use PCR, Human panel I + II (Catalog number: 339322, QIAGEN). The amplification was carried out using a LightCyclerR 480 Real-Time PCR System (Roche, Basel, Switzerland) and data were analyzed using Roche LC software 4 (Basel, Switzerland). The ΔCq values were obtained by using the global mean normalization approach to correct all Cq data. Fold-change analysis was performed using 2 × |ΔΔCq| calculation, with ΔΔCq obtained from (∆Cq × T1DM) − (∆Cq × HCs).

### 2.6. IGF-1 and IGFBP-3 Enzyme-Linked Immunosorbent Assay (ELISA)

IGF-1 and IGFBP-3 levels were measured from T1DM and HC serum samples that were obtained from the MERIT study using the Human IGF-I/IGF-1 Quantikine^®^ ELISA Kit (R & D Systems, Minneapolis, MN, USA) and Human IGFBP-3 Quantikine^®^ ELISA Kit (R & D Systems), respectively, according to the protocol set by the manufacturer. Serum samples underwent a 100-fold dilution in Calibrator Diluent RD5-18. SpectraMax^®^ 190 absorbance plate reader (Molecular Devices, San Jose, CA, USA) and SoftMax^®^ Pro Software 5.4 (Molecular Devices) were used to analyze the data from both the IGF-1 and the IGFBP-3 assays. A standard curve was created by generating a log/log curve fit. Both the IGF-1 and the IGFBP-3 assays were read at 450 nm with correction at 570 nm.

### 2.7. Ingenuity Pathway Analysis (IPA) of miR-106b-3p and Its mRNA Targets

Analyses of miR-106b-3p’s predicted downstream pathways, targets, and cellular functions and their associations with CVD were performed in this study using Ingenuity Pathway Analysis (IPA) software 9.0 (Ingenuity, Redwood City, CA, USA). Expression data of miR-106b-3p were input into the software. Ingenuity Knowledge Base (IKB) is used by IPA and, in this study, helped create a network between miR-106b-3p and other molecules and diseases based on weak or strong evidence. The interaction sites of miR-106b-3p were predicted via the microRNA target filter tool, which used TargetScanHuman release 8.0 (https://www.targetscan.org/vert_80/ accessed on 14 December 2023) and Diana-TarBase v8 (https://dianalab.e-ce.uth.gr/html/diana/web/index.php?r=tarbasev8 accessed on 14 December 2023) databases.

### 2.8. Statistical Analysis

Data were displayed as mean ± standard deviation (SD). GraphPad Prism 9.0 (GraphPad Software, San Diego, CA, USA) was used to carry out statistical analyses, with statistically significant results being indicated by a *p*-value of less than 0.05. Shapiro–Wilk tests were performed to assess the normality of the data. Comparisons between groups used one-way ANOVA with Tukey test, unpaired Student *t*-tests, or Mann–Whitney tests, and within-group comparisons used paired Student *t*-tests or Wilcoxon signed rank tests, depending on the distribution. A linear regression analysis was used to examine any correlations between miR-106b-3p, IGF-1, IGFBP-3, and other factors.

## 3. Results

### 3.1. Characteristics of Studied Subjects

The participant data analyzed in this study were obtained from the 29 T1DM patients and 20 age- and gender-matched healthy controls (HCs) recruited into the MERIT study. T1DM patients’ inclusion criteria included HbA1c < 8.5% (69 mmol/mmol) and no presence of diabetic complications such as evident CVD. Participant characteristics were previously summarized [48]. T1DM participants were administered metformin for 8 weeks to the highest tolerated dose or with a dose titrated up to a maximum of 1 g twice a day.

### 3.2. Comparison of Vascular Health and Inflammatory Markers between HCs and T1DM

We have previously published data from the MERIT study comparing baseline markers of vascular health and inflammation between HC participants and T1DM participants, using unpaired *t*-tests or Mann–Whitney U tests. There were significantly higher levels of IL-7 (*p* = 0.008), IL-8 (*p* = 0.003), IL-10 (*p* = 0.008), VEGF-C (*p* = 0.013), VEGF-D (*p* = 0.002), CRP (*p* < 0.001), TNF-α (*p* = 0.041), and thrombomodulin (*p* = 0.046) in T1DM compared to HCs. The study showed significantly lower levels of CFU-Hills (*p* = 0.04), FAA (*p* = 0.017), cEPC/CD45dimCD34 + VEGFR-2+ + cells (*p* < 0.001), CD34+/100 lymphocytes (*p* < 0.001), PACs (*p*  <  0.001) (7, 8, 10, 39), and sICAM-1 (*p* = 0.002). We have recently reported that T1DM is a chronic inflammatory state [49].

### 3.3. Comparison of IGF-1 and IGFBP-3 Levels in HCs and T1DM and the Effects of Metformin

**Figure 1:**
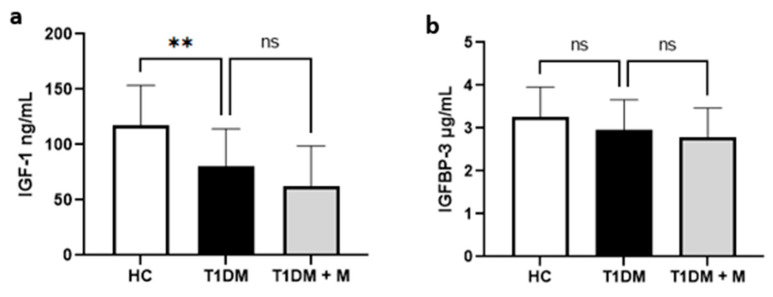
The comparison between HC, T1DM (before metformin), and T1DM + M (after metformin) levels of (**a**) IGF-1 (ng/mL), and (**b**) IGFBP-3 (μg/mL). Data are presented as means ± SD and the difference between groups is analyzed by one-way ANOVA with a Tukey test; ** *p* < 0.01; HC: healthy controls; IGF: insulin-like growth factor; IGFBP: insulin-like growth factor binding protein; T1DM: type 1 diabetic mellitus.

Analysis of ELISA data showed that IGF-1 levels were significantly lower at baseline in T1DM compared to HCs (*p* = 0.0015, Figure 1a). However, IGF-1 levels were not significantly different in T1DM participants before and after metformin administration (*p* = 0.2304, Figure 1a). There was no significant difference in baseline levels of IGFBP-3 between HCs and T1DM (*p* = 0.339, Figure 1b). There was also no significant difference in IGFBP-3 levels in T1DM participants before and after metformin therapy (*p* = 0.712, Figure 1b).

### 3.4. IGF-1 Correlations with Inflammatory and Vascular Health Markers

**Figure 2:**
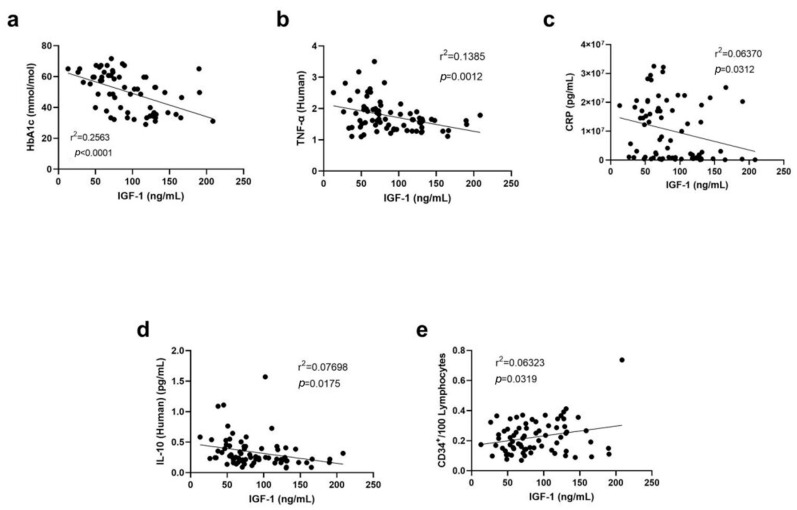
Correlations between IGF-1 levels and levels of (**a**) HbA1c, (**b**) TNF-α, (**c**) CRP (pg/mL), (**d**) IL-10, and (**e**) CD34+/100 lymphocytes. Linear regression analyses were performed to assess the correlations. CD: cluster of differentiation; CRP: c-reactive protein; HbA1c: glycated hemoglobin; IGF: insulin-like growth factor; IL: interleukin; TNF-α: tumor necrosis factor-alpha.

Linear regression analysis determined that IGF-1 levels were significantly negatively correlated with HbA1c (*p* < 0.0001, Figure 2a), TNF-α (*p* = 0.0012, Figure 2b), CRP (*p* = 0.0312, Figure 2c), and IL-10 (*p* = 0.0175, Figure 2d), which are markers of inflammation. However, no significant correlation was detected with the other inflammatory markers including IL-7, IL-8, VEGF-C, VEGF-D, and thrombomodulin. IGF-1 levels were also found to be positively correlated with the vascular health marker CD34+/100 lymphocytes (*p* = 0.0319, Figure 2e).

### 3.5. Comparisons of miR-106b-3p Expression in HCs and T1DM and the Effects of Metformin

**Figure 3:**
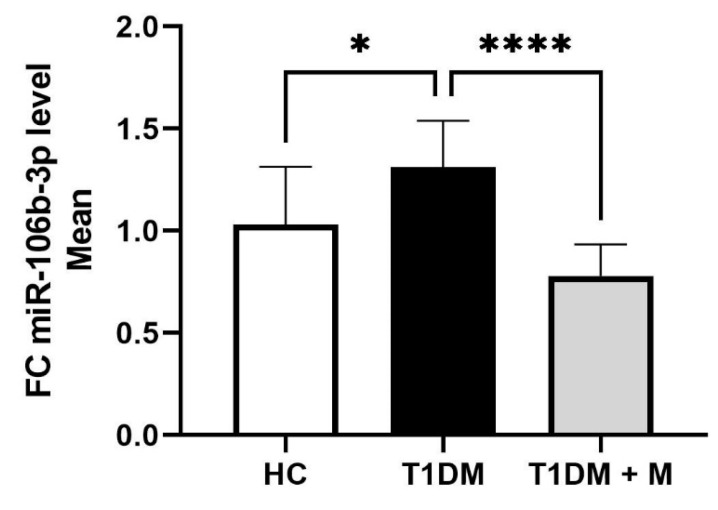
Comparisons of the expression of miR-106b-3p in CFU-Hill colonies in HC, T1DM (before metformin), and T1DM + M (after metformin). Data are presented as means ± SD and the difference between groups is analyzed by one-way ANOVA followed by a Tukey test; * *p* < 0.05; **** *p* < 0.0001. FC: fold change; HC: healthy control; T1DM: type 1 diabetes mellitus.

miR-106b-3p expression was found to be significantly upregulated in CFU-Hills in T1DM compared to HCs (*p* = 0.0162, Figure 3), while the expression of miR-106b-3p expression in CFU-Hills was significantly downregulated after metformin therapy (*p* < 0.0001, Figure 3).

### 3.6. IGF-1 and IGFBP-3 Correlations with miR-106b-3p

**Figure 4:**
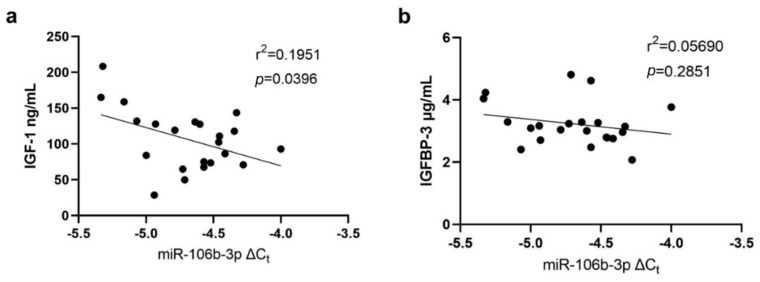
Correlation between miR-106b-3p expression in CFU-Hill colonies and levels of (**a**) IGF-1 (ng/mL), and (**b**) IGFBP-3 (μg/mL). Correlations were assessed using linear regression analyses. Ct: cycle threshold; IGF: insulin-like growth factor; IGFBP: insulin-like growth factor binding protein.

Linear regression analysis found that IGF-1 levels were significantly negatively correlated with miR-106b-3p expression levels in CFU-Hill colonies (*p* = 0.0396, Figure 4a). Levels of IGFBP-3 were not found to be significantly correlated with miR-106b-3p expression levels in CFU-Hill colonies (*p* = 0.2851, Figure 4b).

### 3.7. miR-106b-3p Correlations with Vascular Health Markers

**Figure 5:**
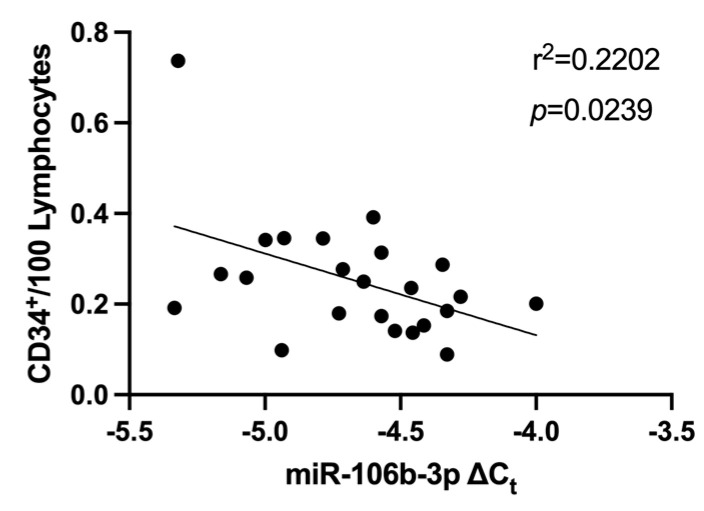
Comparison of miR-106b-3p expression in CFU-Hill colonies with the levels of CD34+/100 lymphocytes Correlations were assessed using linear regression analyses. CD: cluster of differentiation; Ct: cycle threshold.

Linear regression analysis showed that miR-106b-3p expression levels in CFU-Hills were significantly negatively correlated with vascular health marker CD34+/100 lymphocytes (*p* = 0.0239, Figure 5). No significant correlation between miR-106b-3p was detected between the other studied inflammatory or vascular makers; CFU-Hills, cEPC, PAC, FAA, and sICAM-1.

### 3.8. Ingenuity Pathway Analysis (IPA) of miR-106b-3p

The knowledge-based database IPA software 9 was used to predict the downstream targets of miR-106b-3p in connection with CVD development. Upregulated miR-106b-3p was input into the IPA software. This resulted in miR-106b-3p being predicted as anti-angiogenic, anti-prolific, pro-inflammatory, and associated with increased risk of diabetes mellitus and CVD.

**Figure 6:**
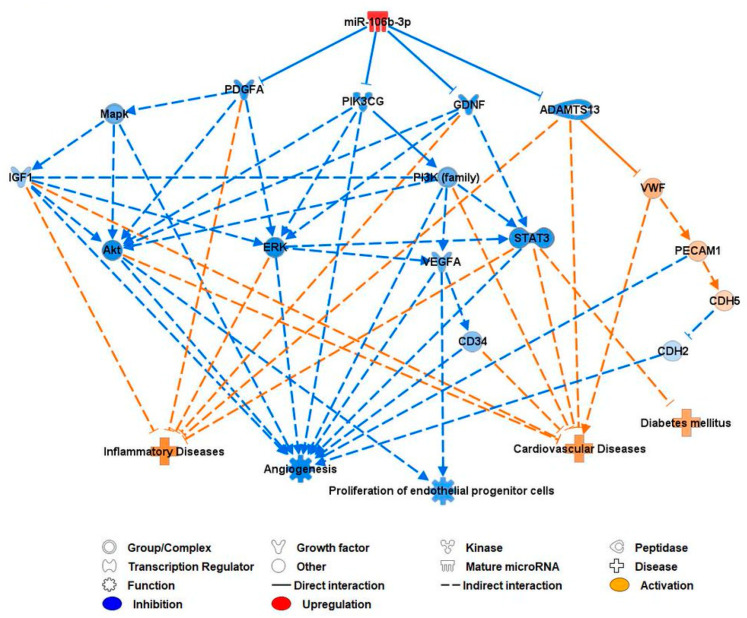
IPA prediction network of miR-106b-3p and its molecular targets and pathways relating to CVD. ADAMTS13: a disintegrin and metalloprotease with thrombospondin type 1 repeats 13; Akt: Ak strain transforming; CD: cluster of differentiation; CDH: cadherin; ERK: extracellular signal-regulated kinases; GDNF: glial-cell-line-derived neurotrophic factor; IGF: insulin-like growth factor; MAPK: mitogen-activated protein kinase; PDGFA: platelet-derived growth factor subunit A; PECAM1: platelet endothelial cell adhesion molecule 1; PI3K: phosphoinositide 3-kinase; PIK3CG: phosphatidylinositol-3-kinase catalytic subunit gamma; STAT3: signal transducer and activator of transcription 3; vWF: von Willebrand factor; VEGF: vascular endothelial growth factor.

IPA predicted miR-106b-3p to inhibit the expression of PDGFA, PIK3CG, GDNF, and ADAMTS13. These in turn were predicted to inhibit MAPK, ERK, Akt, the PI3K family, STAT3, VEGFA, IGF-1, CDH2, and CD34, and activate vWF, PECAM1 (CD31), and CDH5 (CD144). These all led to the predicted inhibition of angiogenesis and EPC proliferation, and activation of inflammatory disease, diabetes mellitus, and CVD (Figure 6).

### 3.9. Ingenuity Pathway Analysis (IPA) of miR-106b-3p after Metformin Therapy

Metformin was predicted to indirectly inhibit miR-106b-3p, which resulted in predicted pro-angiogenic, prolific, and anti-inflammatory effects with reduced risk of diabetes mellitus and CVD. Metformin was also predicted to indirectly activate IGF-1 and inhibit inflammatory diseases, diabetes mellitus, and CVD through other pathways unrelated to miR-106b-3p.

**Figure 7:**
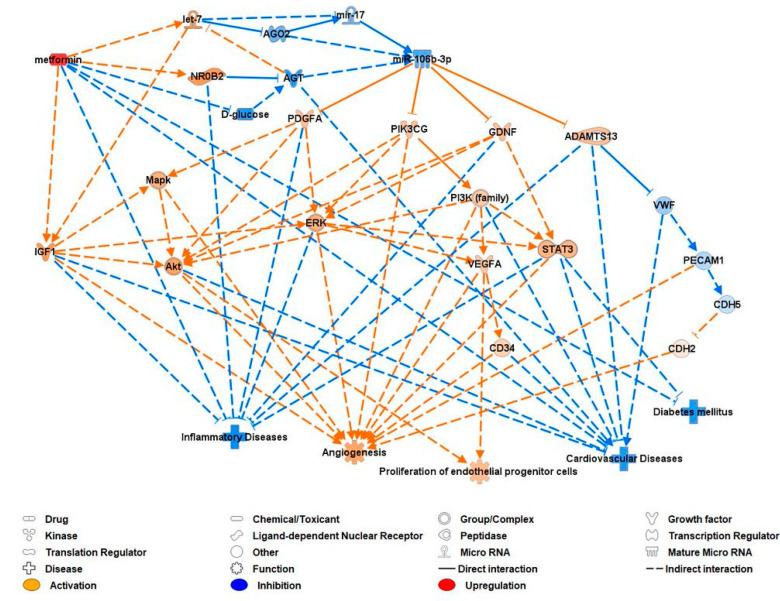
IPA prediction network of miR-106b-3p and its molecular targets and pathways relating to CVD after metformin therapy. ADAMTS13: a disintegrin and metalloprotease with thrombospondin type 1 repeats 13; AGO2: argonaute 2; AGT: angiotensinogen; Akt: Ak strain transforming; CD: cluster of differentiation; CDH: cadherin; D-glucose: dextrorotatory glucose; ERK: extracellular signal-regulated kinases; GDNF: glial-cell-line-derived neurotrophic factor; let-7: lethal-7; NR0B2: nuclear receptor subfamily 0 group B member 2; PDGFA: platelet-derived growth factor subunit A; PECAM1: platelet endothelial cell adhesion molecule 1; PI3K: phosphoinositide 3-kinase; PIK3CG: phosphatidylinositol-3-kinase catalytic subunit gamma; STAT3: signal transducer and activator of transcription 3; VWF: von Willebrand factor; VEGFA: vascular endothelial growth factor A.

The inhibition of miR-106b-3p by metformin was predicted to occur via indirect activation of let-7 and NR0B2, and inhibition of D-glucose, AGO2, AGT, and mir-17. This downregulation of miR-106b-3p was predicted to activate the expression of PDGFA, PIK3CG, GDNF, AND ADAMTS13. These in turn were predicted to activate MAPK, ERK, Akt, the PI3K family, STAT3, VEGFA, IGF-1, CDH2, and CD34, and inhibit VWF, PECAM1 (CD31), and CDH5 (CD144). These all led to the predicted activation of angiogenesis and proliferation of EPCs, and inhibition of inflammatory disease, diabetes mellitus, and CVD (Figure 7).

## 4. Discussion

This study is the first to investigate the expression of miR-106b-3p in CFU-Hill colonies, its correlations with vascular health markers, and the effects of metformin in T1DM as a model of subclinical CVD. It also investigated IGF-1 levels and their correlations with vascular health markers, as well as the effects of metformin in this model. It shows significantly upregulated levels of sICAM-1, an inflammatory-stimulated adhesion molecule, and decreased levels of pro-angiogenic IGF-1 in T1DM versus HCs. Research has associated sICAM-1 with increased CVD risk [50] and indicated that high concentrations may be an early biomarker of atherosclerosis [51]. This supports the use of T1DM as a model of subclinical CVD.

### 4.1. Reduced IGF-1 and IGFBP-3 Levels in T1DM and the Effects of Metformin

The results of this study showed that IGF-1 levels were significantly lower in T1DM compared to HCs. This is in accordance with other studies showing reduced serum IGF-1 levels in those with diabetes [52,53] and those with CVD [27,54,55,56]. IGF-1 is known to promote cell proliferation and have anti-inflammatory and anti-apoptotic effects through the PI3K/Akt/mTOR and Ras/Raf/MEK/ERK pathways. This has a role in regulating cardiovascular function. A cohort study found that reduced IGF-1 levels were associated with acute coronary syndrome (ACS) development, and increased levels were associated with a lower risk of developing MI [54]. Similarly, low IGF-1 levels have been correlated with atherosclerosis as well as a higher CVD mortality risk [56]. Low IGF-1 levels have also been associated with increased levels of pro-inflammatory cytokines such as TNF-α, which are observed in the chronic inflammatory states seen in CVD [52]. This highlights the apparent involvement of IGF-1 signaling in CVD pathogenesis and demonstrates that low IGF-1 levels could be used as a potential indicator of CVD development in T1DM.

IGFBP-3 levels were not significantly different between the HCs and T1DM participants. Other researchers showed reduced IGFBP-3 in T1DM and CVD, but these studies were not in well-controlled T1DM cohorts [57]. As IGFBP-3 carries the majority of circulating IGF-1 [27], it was expected that low IGF-1 levels would coincide with low IGFBP-3 levels. However, the lower IGF-1 levels in T1DM, despite the lack of difference in IGFBP-3, could be due to changes in levels of other IGFBPs.

Metformin therapy had no significant impact on IGF-1 or IGFBP-3 levels in T1DM patients. However, although the levels were unchanged, it does not indicate the lack of metformin’s effect on the bioactivity of IGF-1 and IGFBP-3. A placebo-controlled study found that a reduction in IGF-1 bioavailability did not affect total IGF-1 and IGFBP-3 levels [58]. A systemic analysis and meta-analysis of randomized controlled trials showed that the effect of metformin on serum IGF-1 levels is discordant depending on the age of individuals, dose–response, and duration of treatment [27].

### 4.2. Associations between IGF-1 and Inflammatory and Vascular Health Markers

IGF-1 was significantly negatively correlated with inflammatory markers TNF-α, CRP, and IL-10 and with HbA1c. It was also positively correlated with vascular health marker CD34+/100 lymphocytes. As IGF-1 was shown to be lower in T1DM, this is in accordance with previous publications showing high TNF-α, CRP, and IL-10 and low CD34+ in T1DM [8,9,10].

### 4.3. Upregulation of miR-106b-3p Expression in T1DM

Expression of miR-106b-3p in CFU-Hill colonies was significantly upregulated in T1DM compared to HCs. There is not currently unanimous agreement on the role of miR-106b-3p in cardiovascular pathology, but various studies show its involvement in mechanisms related to CVD. Studies have also associated its precursor miR-106b with suppression of cell proliferation [59] and angiogenesis [36].

WNT/β catenin signaling has been shown to be influenced by miR-106b-3p [60], with multiple studies linking cardiovascular pathology with dysregulation of WNT signaling [61]. Upregulated miR-106b-3p was also shown to downregulate deleted in liver cancer 1 (DLC1) expression [62]. DLC1 is highly expressed in the heart, influences cardiovascular development in zebrafish [63], and may be associated with coronary heart disease (CHD) [64].

Additionally, miR-106b lowers ATP-binding cassette transporter A1 (ABCA1) [65]. ABCA1 is significant in CVD prevention [66], as it is an important mediator in cholesterol homeostasis and has antiatherogenic effects associated with its upregulation [67].

In ECs, miR-106b was shown to have anti-angiogenic effects through inhibiting STAT3 [68]. VEGFA expression is likewise affected by miR-106b [69], with upregulation of VEGFA in choroid specimens being stimulated by reduced miR-106b [36]. IPA predictions support these findings by demonstrating that upregulated miR-106b-3p leads to the predicted indirect inhibition of STAT3 and VEGFA leads to indirect activation of CVD.

### 4.4. Associations between miR-106b-3p and Vascular Health Markers

Expression levels of miR-106b-3p were significantly negatively correlated with IGF-1 and vascular health marker CD34+/100 lymphocytes. Low levels of IGF-1 have been shown to have cardio-adverse associations, such as inflammation, as discussed above. These findings are supported by IPA predictions of upregulated miR-106b-3p leading to indirect inhibition of IGF-1 and activation of CVD.

In this study, miR-106b-3p was negatively correlated with CD34+/100 lymphocytes. As miR-106b-3p was shown to be significantly upregulated in T1DM, this is congruent with studies showing that diabetes is related to reduced levels and performance of CD34+ cells [70,71]. IPA predictions support these findings with upregulated miR-106b-3p being predicted to lead to indirect inhibition of CD34. The negative correlation between miR-106b-3p and CD34 may indicate the anti-angiogenic role of miR-106b-3p in T1DM.

### 4.5. Downregulation of miR-106b-3p Expression Following Metformin Therapy

This study is the first to show that 8-week metformin therapy can significantly downregulate miR-106b-3p in well-controlled T1DM patients to a level comparable to HCs. This is congruent with studies showing that metformin can affect the expression of different miRNAs [8].

Inhibition of miR-106b-3p by metformin was predicted by IPA to occur via nuclear receptor subfamily 0 group B member 2 (NR0B2), angiotensinogen (AGT), lethal-7 (let-7), Argonaute 2 (AGO2), and mir-17 leading to cardioprotective effects. NR0B2 is a unique member of the nuclear receptor superfamily that is crucial in cholesterol metabolism and glucose and inflammation regulation [72]. Studies have shown the anti-atherosclerotic properties of NR0B2 via modification of cholesterol homeostasis and macrophage metabolism [73,74]. Metformin has previously been shown to induce NR0B2 expression [75], which can work as a mechanism to improve glycaemic control [76], but also to influence miRNA expression [77].

Predicted metformin-induced indirect activation of NR0B2 led to inhibition of AGT. AGT is a precursor to angiotensin peptides and a component of the renin–angiotensin system (RAS), which regulates blood pressure and fluid homeostasis [78]. There are many studies linking RAS to CVD development [79], and it has been demonstrated that the reduction of AGT can reduce atherosclerosis [80]. Metformin’s indirect inhibition of d-glucose also led to the inhibition of AGT [81].

Metformin and the inhibition of AGT induced the activation of let-7, a family of miRNAs, which led to the inhibition of miR-17 and AGO2. The miR-17 precursor family contains miR-106b [82], thus its reduction would lead to the downregulation of miR-106b-3p. AGO2 cleavage is required for the production and processing of functional miRNA [83], and the binding of AGO2 to miRNAs increases their stability and extends their half-life [84]. Thus, the metformin-induced inhibition of AGO2 may have decreased the generation and stability of miR-106b-3p, resulting in its downregulation.

These findings support previous studies showing the cardioprotective effects of metformin via mechanisms independent of glycemic change [40,44,85]. It also identifies key targets, which lead to the reduction of cardio-adverse miR-106b-3p, which could be utilized for CVD prevention.

### 4.6. Contribution and Causation

The potential contribution or causality created from miR-106b-3p target gene binding sites was distinguished using the correlation analysis from this study (Table S1). Likely target genes of interest for miR-106b-3p were searched using the TargetScanHuman 8.0 and Diana-TarBase v8 databases, and IPA was employed to find the aptest canonical pathways related to the target genes. Four pertinent target genes (ADAMTS13, GDNF, PDGFA, and PIK3CG) were found to have multiple binding sites with miR-106b-3p. This demonstrates that miR-106b-3p has a potential causal role in increasing inflammation and reducing EPC proliferation and angiogenesis leading to CVD development via these target genes.

### 4.7. Limitations

The potential limitation of our experimental approach is the difficulty in obtaining a sufficient quantity of RNA from CFU-Hill colonies for parallel analysis of mRNA and miRNA in individual subjects. This relatively small sample study requires further validation.

## 5. Conclusions

This study established that IGF-1 was downregulated in T1DM as a subclinical CVD model, was negatively correlated with inflammatory markers, and was positively correlated with vascular health markers. No significant difference in IGFBP-3 between T1DM and HCs was observed, and metformin did not have a significant impact on IGF-1 or IGFBP-3 levels. It was detected that miR-106b-3p was significantly upregulated in CFU-Hills in T1DM and correlated with adverse vascular health markers. IPA also predicted that miR-106b-3p upregulation would lead to CVD development. Metformin was shown to significantly downregulate miR-106b-3p, demonstrating its cardioprotective function.

These findings indicate the possible roles of low IGF-1 and upregulated miR-106b-3p in CVD development. Therefore, they could be used as potential future biomarkers of CVD development or targets for CVD prevention. The miR-106b-3p target genes PDGFA, PIK3CG, GDNF, and ADAMTS13 identified could also be potential future targets for miRNA-based CVD research. Ongoing research is needed to fully understand the roles of IGF-1, miR-106b-3p, and metformin in CVD risk and to validate the findings of this study.

## Data Availability

All data are contained within the article and Supplementary Material.

## Supplementary Materials

The following supporting information can be downloaded at: https://www.mdpi.com/article/10.3390/biomedicines12010171/s1, Table S1. The predicted consequential pairing of miR-106b-3p and transcript target regions.

## Trial Registration

ISRCTN26092132. Available online: http://www.controlled-trials.com (accessed on 13 July 2012).

## References

[1] World Heart Federation (2023). World Heart Report 2023: Confronting the World’s Number One Killer.

[2] World Health Organisation (WHO) Cardiovascular Diseases 2021. https://www.who.int/news-room/fact-sheets/detail/cardiovascular-diseases-(cvds).

[3] Livingstone S.J., Levin D., Looker H.C., Lindsay R.S., Wild S.H., Joss N., Leese G., Leslie P., McCrimmon R.J., Metcalfe W. (2015). Estimated life expectancy in a Scottish cohort with type 1 diabetes, 2008–2010. JAMA.

[4] Lind M., Svensson A.M., Kosiborod M., Gudbjornsdottir S., Pivodic A., Wedel H., Dahlqvist S., Clements M., Rosengren A. (2014). Glycemic control and excess mortality in type 1 diabetes. N. Engl. J. Med..

[5] Ahmed F.W., Rider R., Glanville M., Narayanan K., Razvi S., Weaver J.U. (2016). Metformin improves circulating endothelial cells and endothelial progenitor cells in type 1 diabetes: MERIT study. Cardiovasc. Diabetol..

[6] Asicioglu E., Gogas Yavuz D., Koc M., Ozben B., Yazici D., Deyneli O., Akalin S. (2010). Circulating endothelial cells are elevated in patients with type 1 diabetes mellitus. Eur. J. Endocrinol..

[7] Hill J.M., Zalos G., Halcox J.P., Schenke W.H., Waclawiw M.A., Quyyumi A.A., Finkel T. (2003). Circulating endothelial progenitor cells, vascular function, and cardiovascular risk. N. Engl. J. Med..

[8] Phowira J., Ahmed F.W., Bakhashab S., Weaver J.U. (2022). Upregulated miR-18a-5p in Colony Forming Unit-Hill’s in Subclinical Cardiovascular Disease and Metformin Therapy; MERIT Study. Biomedicines.

[9] Ray S.L., Coulson D.J., Yeoh M.L.Y., Tamara A., Latief J.S., Bakhashab S., Weaver J.U. (2020). The Role of miR-342 in Vascular Health. Study in Subclinical Cardiovascular Disease in Mononuclear Cells, Plasma, Inflammatory Cytokines and PANX2. Int. J. Mol. Sci..

[10] Sibal L., Aldibbiat A., Agarwal S.C., Mitchell G., Oates C., Razvi S., Weaver J.U., Shaw J.A., Home P.D. (2009). Circulating endothelial progenitor cells, endothelial function, carotid intima-media thickness and circulating markers of endothelial dysfunction in people with type 1 diabetes without macrovascular disease or microalbuminuria. Diabetologia.

[11] Boos C.J., Lip G.Y., Blann A.D. (2006). Circulating endothelial cells in cardiovascular disease. J. Am. Coll. Cardiol..

[12] Blann A.D., Woywodt A., Bertolini F., Bull T.M., Buyon J.P., Clancy R.M., Haubitz M., Hebbel R.P., Lip G.Y., Mancuso P. (2005). Circulating endothelial cells. Biomarker of vascular disease. Thromb. Haemost..

[13] Lee K.W., Lip G.Y., Tayebjee M., Foster W., Blann A.D. (2005). Circulating endothelial cells, von Willebrand factor, interleukin-6, and prognosis in patients with acute coronary syndromes. Blood.

[14] Martinez-Sales V., Sanchez-Lazaro I., Vila V., Almenar L., Contreras T., Reganon E. (2011). Circulating endothelial cells in patients with heart failure and left ventricular dysfunction. Dis. Markers.

[15] He X.Y., Chen Z.Z., Cai Y.Q., Xu G., Shang J.H., Kou S.B., Li M., Zhang H.T., Duan C.Z., Zhang S.Z. (2011). Expression of cytokines in rat brain with focal cerebral ischemia after grafting with bone marrow stromal cells and endothelial progenitor cells. Cytotherapy.

[16] Hu C.H., Li Z.M., Du Z.M., Zhang A.X., Rana J.S., Liu D.H., Yang D.Y., Wu G.F. (2010). Expanded human cord blood-derived endothelial progenitor cells salvage infarcted myocardium in rats with acute myocardial infarction. Clin. Exp. Pharmacol. Physiol..

[17] Yoder M.C. (2012). Human endothelial progenitor cells. Cold Spring Harb. Perspect. Med..

[18] Chu K., Jung K.H., Lee S.T., Park H.K., Sinn D.I., Kim J.M., Kim D.H., Kim J.H., Kim S.J., Song E.C. (2008). Circulating endothelial progenitor cells as a new marker of endothelial dysfunction or repair in acute stroke. Stroke.

[19] Heinisch P.P., Bello C., Emmert M.Y., Carrel T., Dressen M., Horer J., Winkler B., Luedi M.M. (2022). Endothelial Progenitor Cells as Biomarkers of Cardiovascular Pathologies: A Narrative Review. Cells.

[20] Werner N., Kosiol S., Schiegl T., Ahlers P., Walenta K., Link A., Bohm M., Nickenig G. (2005). Circulating endothelial progenitor cells and cardiovascular outcomes. N. Engl. J. Med..

[21] Asahara T., Murohara T., Sullivan A., Silver M., van der Zee R., Li T., Witzenbichler B., Schatteman G., Isner J.M. (1997). Isolation of putative progenitor endothelial cells for angiogenesis. Science.

[22] Boppart M.D., De Lisio M., Witkowski S. (2015). Exercise and Stem Cells. Prog. Mol. Biol. Transl. Sci..

[23] Piatkowski A., Grieb G., Simons D., Bernhagen J., van der Hulst R.R. (2013). Endothelial progenitor cells--potential new avenues to improve neoangiogenesis and reendothelialization. Int. Rev. Cell Mol. Biol..

[24] Ghani U., Shuaib A., Salam A., Nasir A., Shuaib U., Jeerakathil T., Sher F., O’Rourke F., Nasser A.M., Schwindt B. (2005). Endothelial progenitor cells during cerebrovascular disease. Stroke.

[25] Laron Z. (2001). Insulin-like growth factor 1 (IGF-1): A growth hormone. Mol. Pathol..

[26] Sarfstein R., Friedman Y., Attias-Geva Z., Fishman A., Bruchim I., Werner H. (2013). Metformin downregulates the insulin/IGF-I signaling pathway and inhibits different uterine serous carcinoma (USC) cells proliferation and migration in p53-dependent or -independent manners. PLoS ONE.

[27] Yang X., Kord-Varkaneh H., Talaei S., Clark C.C.T., Zanghelini F., Tan S.C., Zarezadeh M., Mousavi S.M., Rahmani J., Zhang Y. (2020). The influence of metformin on IGF-1 levels in humans: A systematic review and meta-analysis. Pharmacol. Res..

[28] Laughlin G.A., Barrett-Connor E., Criqui M.H., Kritz-Silverstein D. (2004). The prospective association of serum insulin-like growth factor I (IGF-I) and IGF-binding protein-1 levels with all cause and cardiovascular disease mortality in older adults: The Rancho Bernardo Study. J. Clin. Endocrinol. Metab..

[29] Kooijman R. (2006). Regulation of apoptosis by insulin-like growth factor (IGF)-I. Cytokine Growth Factor Rev..

[30] Assmann T.S., Recamonde-Mendoza M., De Souza B.M., Crispim D. (2017). MicroRNA expression profiles and type 1 diabetes mellitus: Systematic review and bioinformatic analysis. Endocr. Connect..

[31] Miao C., Chang J., Zhang G., Fang Y. (2018). MicroRNAs in type 1 diabetes: New research progress and potential directions. Biochem. Cell Biol..

[32] Zhang Y., Zhao Y.P., Gao Y.F., Fan Z.M., Liu M.Y., Cai X.Y., Xia Z.K., Gao C.L. (2015). Silencing miR-106b improves palmitic acid-induced mitochondrial dysfunction and insulin resistance in skeletal myocytes. Mol. Med. Rep..

[33] Sun X., Chen G., Xie Y., Jiang D., Han J., Chen F., Song Y. (2020). Qiliqiangxin improves cardiac function and attenuates cardiac remodelling in doxorubicin-induced heart failure rats. Pharm. Biol..

[34] Siwaponanan P., Kaewkumdee P., Phromawan W., Udompunturak S., Chomanee N., Udol K., Pattanapanyasat K., Krittayaphong R. (2022). Increased expression of six-large extracellular vesicle-derived miRNAs signature for nonvalvular atrial fibrillation. J. Transl. Med..

[35] Zhang J., Ren J., Chen H., Geng Q. (2014). Inflammation induced-endothelial cells release angiogenesis associated-microRNAs into circulation by microparticles. Chin. Med. J. (Engl.).

[36] Menard C., Wilson A.M., Dejda A., Miloudi K., Binet F., Crespo-Garcia S., Parinot C., Pilon F., Juneau R., Andriessen E.M. (2020). miR-106b suppresses pathological retinal angiogenesis. Aging.

[37] UK Prospective Diabetes Study (UKPDS) Group (1998). Effect of intensive blood-glucose control with metformin on complications in overweight patients with type 2 diabetes (UKPDS 34). Lancet.

[38] Fung C.S., Wan E.Y., Wong C.K., Jiao F., Chan A.K. (2015). Effect of metformin monotherapy on cardiovascular diseases and mortality: A retrospective cohort study on Chinese type 2 diabetes mellitus patients. Cardiovasc. Diabetol..

[39] Li S.N., Wang X., Zeng Q.T., Feng Y.B., Cheng X., Mao X.B., Wang T.H., Deng H.P. (2009). Metformin inhibits nuclear factor kappaB activation and decreases serum high-sensitivity C-reactive protein level in experimental atherogenesis of rabbits. Heart Vessel..

[40] Oliveira P.W.C., de Sousa G.J., Birocale A.M., Gouvea S.A., de Figueiredo S.G., de Abreu G.R., Bissoli N.S. (2020). Chronic metformin reduces systemic and local inflammatory proteins and improves hypertension-related cardiac autonomic dysfunction. Nutr. Metab. Cardiovasc. Dis..

[41] Pitocco D., Zaccardi F., Tarzia P., Milo M., Scavone G., Rizzo P., Pagliaccia F., Nerla R., Di Franco A., Manto A. (2013). Metformin improves endothelial function in type 1 diabetic subjects: A pilot, placebo-controlled randomized study. Diabetes Obes. Metab..

[42] Salvatore T., Pafundi P.C., Galiero R., Rinaldi L., Caturano A., Vetrano E., Aprea C., Albanese G., Di Martino A., Ricozzi C. (2020). Can Metformin Exert as an Active Drug on Endothelial Dysfunction in Diabetic Subjects?. Biomedicines.

[43] Yin M., van der Horst I.C., van Melle J.P., Qian C., van Gilst W.H., Sillje H.H., de Boer R.A. (2011). Metformin improves cardiac function in a nondiabetic rat model of post-MI heart failure. Am. J. Physiol. Heart Circ. Physiol..

[44] Paiva M., Riksen N.P., Davidson S.M., Hausenloy D.J., Monteiro P., Goncalves L., Providencia L., Rongen G.A., Smits P., Mocanu M.M. (2009). Metformin prevents myocardial reperfusion injury by activating the adenosine receptor. J. Cardiovasc. Pharmacol..

[45] Liu Y., Tang G., Zhang Z., Wang Y., Yang G.Y. (2014). Metformin promotes focal angiogenesis and neurogenesis in mice following middle cerebral artery occlusion. Neurosci. Lett..

[46] Petrie J.R., Chaturvedi N., Ford I., Brouwers M., Greenlaw N., Tillin T., Hramiak I., Hughes A.D., Jenkins A.J., Klein B.E.K. (2017). Cardiovascular and metabolic effects of metformin in patients with type 1 diabetes (REMOVAL): A double-blind, randomised, placebo-controlled trial. Lancet Diabetes Endocrinol..

[47] Arunachalam G., Lakshmanan A.P., Samuel S.M., Triggle C.R., Ding H. (2016). Molecular Interplay between microRNA-34a and Sirtuin1 in Hyperglycemia-Mediated Impaired Angiogenesis in Endothelial Cells: Effects of Metformin. J. Pharmacol. Exp. Ther..

[48] Tamara A., Coulson D.J., Latief J.S., Bakhashab S., Weaver J.U. (2021). Upregulated anti-angiogenic miR-424-5p in type 1 diabetes (model of subclinical cardiovascular disease) correlates with endothelial progenitor cells, CXCR1/2 and other parameters of vascular health. Stem Cell Res. Ther..

[49] West D.J., Campbell M.D., Gonzalez J.T., Walker M., Stevenson E.J., Ahmed F.W., Wijaya S., Shaw J.A., Weaver J.U. (2015). The inflammation, vascular repair and injury responses to exercise in fit males with and without Type 1 diabetes: An observational study. Cardiovasc. Diabetol..

[50] Jenny N.S., Arnold A.M., Kuller L.H., Sharrett A.R., Fried L.P., Psaty B.M., Tracy R.P. (2006). Soluble intracellular adhesion molecule-1 is associated with cardiovascular disease risk and mortality in older adults. J. Thromb. Haemost..

[51] Gross M.D., Bielinski S.J., Suarez-Lopez J.R., Reiner A.P., Bailey K., Thyagarajan B., Carr J.J., Duprez D.A., Jacobs D.R. (2012). Circulating soluble intercellular adhesion molecule 1 and subclinical atherosclerosis: The Coronary Artery Risk Development in Young Adults Study. Clin. Chem..

[52] Nambam B., Schatz D. (2018). Growth hormone and insulin-like growth factor-I axis in type 1 diabetes. Growth Horm. IGF Res..

[53] Shapiro M.R., Wasserfall C.H., McGrail S.M., Posgai A.L., Bacher R., Muir A., Haller M.J., Schatz D.A., Wesley J.D., von Herrath M. (2020). Insulin-Like Growth Factor Dysregulation Both Preceding and Following Type 1 Diabetes Diagnosis. Diabetes.

[54] Ruidavets J.B., Luc G., Machez E., Genoux A.L., Kee F., Arveiler D., Morange P., Woodside J.V., Amouyel P., Evans A. (2011). Effects of insulin-like growth factor 1 in preventing acute coronary syndromes: The PRIME study. Atherosclerosis.

[55] Shai S.Y., Sukhanov S., Higashi Y., Vaughn C., Rosen C.J., Delafontaine P. (2011). Low circulating insulin-like growth factor I increases atherosclerosis in ApoE-deficient mice. Am. J. Physiol. Heart Circ. Physiol..

[56] Ungvari Z., Csiszar A. (2012). The emerging role of IGF-1 deficiency in cardiovascular aging: Recent advances. J. Gerontol. A Biol. Sci. Med. Sci..

[57] Yeap B.B., Chubb S.A., McCaul K.A., Ho K.K., Hankey G.J., Norman P.E., Flicker L. (2011). Associations of IGF1 and IGFBPs 1 and 3 with all-cause and cardiovascular mortality in older men: The Health In Men Study. Eur. J. Endocrinol..

[58] Sarem Z., Bumke-Vogt C., Mahmoud A.M., Assefa B., Weickert M.O., Adamidou A., Bahr V., Frystyk J., Mohlig M., Spranger J. (2017). Glucagon Decreases IGF-1 Bioactivity in Humans, Independently of Insulin, by Modulating Its Binding Proteins. J. Clin. Endocrinol. Metab..

[59] Liu L., Chen H., Jiang T., He D. (2022). MicroRNA-106b overexpression suppresses synovial inflammation and alleviates synovial damage in patients with rheumatoid arthritis. Mod. Rheumatol..

[60] Qiao G., Dai C., He Y., Shi J., Xu C. (2019). Effects of miR-106b-3p on cell proliferation and epithelial-mesenchymal transition, and targeting of ZNRF3 in esophageal squamous cell carcinoma. Int. J. Mol. Med..

[61] Foulquier S., Daskalopoulos E.P., Lluri G., Hermans K.C.M., Deb A., Blankesteijn W.M. (2018). WNT Signaling in Cardiac and Vascular Disease. Pharmacol. Rev..

[62] Liu H., Liu Y., Sun P., Leng K., Xu Y., Mei L., Han P., Zhang B., Yao K., Li C. (2020). Colorectal cancer-derived exosomal miR-106b-3p promotes metastasis by down-regulating DLC-1 expression. Clin. Sci..

[63] Linnerz T., Bertrand J.Y. (2021). Dlc1 controls cardio-vascular development downstream of Vegfa/Kdrl/Nrp1 signaling in the zebrafish embryo. bioRxiv.

[64] Lin B., Wang Y., Wang Z., Tan H., Kong X., Shu Y., Zhang Y., Huang Y., Zhu Y., Xu H. (2014). Uncovering the rare variants of DLC1 isoform 1 and their functional effects in a Chinese sporadic congenital heart disease cohort. PLoS ONE.

[65] Kim J., Yoon H., Ramirez C.M., Lee S.M., Hoe H.S., Fernandez-Hernando C., Kim J. (2012). MiR-106b impairs cholesterol efflux and increases Abeta levels by repressing ABCA1 expression. Exp. Neurol..

[66] Van Eck M. (2014). ATP-binding cassette transporter A1: Key player in cardiovascular and metabolic disease at local and systemic level. Curr. Opin. Lipidol..

[67] Wang N., Tall A.R. (2003). Regulation and mechanisms of ATP-binding cassette transporter A1-mediated cellular cholesterol efflux. Arterioscler. Thromb. Vasc. Biol..

[68] Maimaiti A., Maimaiti A., Yang Y., Ma Y. (2016). MiR-106b exhibits an anti-angiogenic function by inhibiting STAT3 expression in endothelial cells. Lipids Health Dis..

[69] Nunes D.N., Dias-Neto E., Cardo-Vila M., Edwards J.K., Dobroff A.S., Giordano R.J., Mandelin J., Brentani H.P., Hasselgren C., Yao V.J. (2015). Synchronous down-modulation of miR-17 family members is an early causative event in the retinal angiogenic switch. Proc. Natl. Acad. Sci. USA.

[70] Dore F.J., Domingues C.C., Ahmadi N., Kundu N., Kropotova Y., Houston S., Rouphael C., Mammadova A., Witkin L., Khiyami A. (2018). The synergistic effects of saxagliptin and metformin on CD34+ endothelial progenitor cells in early type 2 diabetes patients: A randomized clinical trial. Cardiovasc. Diabetol..

[71] Fadini G.P., Sartore S., Agostini C., Avogaro A. (2007). Significance of endothelial progenitor cells in subjects with diabetes. Diabetes Care.

[72] Yuk J.M., Jin H.S., Jo E.K. (2016). Small Heterodimer Partner and Innate Immune Regulation. Endocrinol. Metab..

[73] Koelwyn G.J., Corr E.M., Erbay E., Moore K.J. (2018). Regulation of macrophage immunometabolism in atherosclerosis. Nat. Immunol..

[74] Wang L., Lee Y.K., Bundman D., Han Y., Thevananther S., Kim C.S., Chua S.S., Wei P., Heyman R.A., Karin M. (2002). Redundant pathways for negative feedback regulation of bile acid production. Dev. Cell.

[75] Jang W.G., Kim E.J., Bae I.H., Lee K.N., Kim Y.D., Kim D.K., Kim S.H., Lee C.H., Franceschi R.T., Choi H.S. (2011). Metformin induces osteoblast differentiation via orphan nuclear receptor SHP-mediated transactivation of Runx2. Bone.

[76] Kim Y.D., Park K.G., Lee Y.S., Park Y.Y., Kim D.K., Nedumaran B., Jang W.G., Cho W.J., Ha J., Lee I.K. (2008). Metformin inhibits hepatic gluconeogenesis through AMP-activated protein kinase-dependent regulation of the orphan nuclear receptor SHP. Diabetes.

[77] Song Y., Lu S., Zhao J., Wang L. (2017). Nuclear Receptor SHP: A Critical Regulator of miRNA and lncRNA Expression and Function. Nucl. Recept. Res..

[78] Lu H., Cassis L.A., Kooi C.W., Daugherty A. (2016). Structure and functions of angiotensinogen. Hypertens. Res..

[79] Wu C.H., Mohammadmoradi S., Chen J.Z., Sawada H., Daugherty A., Lu H.S. (2018). Renin-Angiotensin System and Cardiovascular Functions. Arterioscler. Thromb. Vasc. Biol..

[80] Xu Y., Rong J., Zhang Z. (2021). The emerging role of angiotensinogen in cardiovascular diseases. J. Cell Physiol..

[81] Satou R., Cypress M.W., Woods T.C., Katsurada A., Dugas C.M., Fonseca V.A., Navar L.G. (2020). Blockade of sodium-glucose cotransporter 2 suppresses high glucose-induced angiotensinogen augmentation in renal proximal tubular cells. Am. J. Physiol. Ren. Physiol..

[82] Xia X., Lu H., Li C., Huang Y., Wang Y., Yang X., Zheng J.C. (2019). miR-106b regulates the proliferation and differentiation of neural stem/progenitor cells through Tp53inp1-Tp53-Cdkn1a axis. Stem Cell Res. Ther..

[83] Cifuentes D., Xue H., Taylor D.W., Patnode H., Mishima Y., Cheloufi S., Ma E., Mane S., Hannon G.J., Lawson N.D. (2010). A novel miRNA processing pathway independent of Dicer requires Argonaute2 catalytic activity. Science.

[84] Laffont B., Corduan A., Plé H., Duchez A.C., Cloutier N., Boilard E., Provost P. (2013). Activated platelets can deliver mRNA regulatory Ago2•microRNA complexes to endothelial cells via microparticles. Blood.

[85] Yin M., Li C., Jiang J., Le J., Luo B., Yang F., Fang Y., Yang M., Deng Z., Ni W. (2021). Cell adhesion molecule-mediated therapeutic strategies in atherosclerosis: From a biological basis and molecular mechanism to drug delivery nanosystems. Biochem. Pharmacol..

