# Supplementary material for: Upregulation of Anti-Angiogenic miR-106b-3p Correlates Negatively with IGF-1 and Vascular Health Parameters in a Model of Subclinical Cardiovascular Disease: Study with Metformin Therapy
Source: Biomedicines. 2024 Jan 12;12(1):171. doi: 10.3390/biomedicines12010171 (PMC10813602; doi:10.3390/biomedicines12010171)
Supplement: Supplementary file 1 [file biomedicines-12-00171-s001.zip › biomedicines-2816472-supplementary.pdf]

Supplementary Material

Upregulation of Anti-Angiogenic miR-106b-3p Correlates Negatively with IGF-1 and Vascular Health Parameters in a Model of Subclinical Cardiovascular Disease: Study with Metformin Therapy

Sherin Bakhashab [1,2,3], Josie O'Neill [2,4], Rosie Barber [2,4], Catherine Arden [4] and Jolanta U. Weaver [2,5,6,*]

1. Biochemistry Department, King Abdulaziz University, P.O. Box 80218, Jeddah 21589, Saudi Arabia; sbakhashab@kau.edu.sa (S.B.)
2. Translational and Clinical Research Institute, Newcastle University, Newcastle upon Tyne NE2 4HH, UK; josieoneill@virginmedia.com (J.O.); rosie_barber@icloud.com (R.B.); Jolanta.Weaver@newcastle.ac.uk (J.U.W)
3. Center of Excellence in Genomic Medicine Research, King Abdulaziz University, Jeddah 2189, Saudi Arabia
4. Biosciences Institute, Newcastle University, Newcastle upon Tyne NE2 4HH, UK; catherine.arden@newcastle.ac.uk (C.A.)
5. Department of Diabetes, Queen Elizabeth Hospital, Gateshead, Newcastle Upon Tyne NE9 6SH, UK
6. Vascular Biology and Medicine Theme, Newcastle University, Newcastle upon Tyne NE1 7RU, UK

* Correspondence: Jolanta.Weaver@newcastle.ac.uk; Tel.: +44-191-445-2181

Table S1. The predicted consequential pairing of miR-106b-3p and transcript target regions.

**Target gene**	**Representative transcript**	**Gene name**	**Transcript position**	**Predicted consequential pairing of target region. Transcript (top) and miRNA (bottom)**	**Site type**
ADAMTS13	ENST00000371916.1	A disintegrin and metalloprotease with thrombospondin type 1 repeats 13	213-219 3' UTR	(transcript) 5'GAGUUCAUGUCGCAA**CAGUGCGC** (miRNA) 3'CGUCGUUCAUGGGU**GUCACGC**C	7mer-m8
ADAMTS13	ENST00000371916.1	A disintegrin and metalloprotease with thrombospondin type 1 repeats 13	1165-1171 3' UTR	(transcript) 5'GCCUGCGGGAGCGGC**CAGUGCG**C (miRNA) 3'CGUCGUUCAUGGGU**GUCACGC**C	7mer-m8
ADAMTS13	ENST00000371916.1	A disintegrin and metalloprotease with thrombospondin type 1 repeats 13	2576-2582 3' UTR	(transcript) 5'GAGUUCUCUCAACUG**CAGUGCG**G (miRNA) 3'CGUCGUUCAUGGGU**GUCACGC**C	7mer-m8
GDNF	ENST00000326524.2	Glial cell derived neurotrophic factor	2049-2055 3' UTR	(transcript) 5'CUCCAGCCUAAGUGA---**CAGUGCG**G (miRNA) 3'CGUCGUUCAUGGGU**GUCACGC**C	7mer-m8
GDNF	ENST00000326524.2	Glial cell derived neurotrophic factor	2601-2608 3' UTR	(transcript) 5'UUCAGAGAACCUUGG**CAGUGCGA** (miRNA) 3'CGUCGUUCAUGGGU**GUCACGC**C	8mer
PDGFA	ENST00000402802.3	Platelet derived growth factor subunit A	753-760 3' UTR	(transcript) 5'CUGUAACUGUCAGGA**CAGUGCGA** (miRNA) 3'CGUCGUUCAUGGGU**GUCACGC**C	8mer
PDGFA	ENST00000354513.5	Platelet derived growth factor subunit A	777-784 3' UTR	(transcript) 5'CUGUAACUGUCAGGA**CAGUGCGA** (miRNA) 3'CGUCGUUCAUGGGU**GUCACGC**C	8mer
PIK3CG	ENST00000359195.3	Phosphatidylinositol-3-kinase catalytic subunit gamma	25-31 3' UTR	(transcript) 5'CUAGAAUCAAAAACA**AGUUAGU**G (miRNA) 3'AGUGAACUUAGACG----**UCAAUCA**U	7mer-m8
PIK3CG	ENST00000359195.3	Phosphatidylinositol-3-kinase catalytic subunit gamma	520-526 3' UTR	(transcript) 5'AAAGCAAGGAAAGCG-**AGUUAGU**C (miRNA) 3'AGUGAACUUAGACG**UCAAUCA**U	7mer-m8

Predictions of interaction sites between miR-106b-3p and the transcripts used the TargetScanHuman 8.0 (https://www.targetscan.org/vert_80/ accessed on 16 June 2023) and Diana-TarBase v8 (https://dianalab.e-ce.uth.gr/html/diana/web/index.php?r=tarbasev8 accessed on 16 June 2023) databases. Predicted consequential pairing of miR-106b-3p and transcript target region are indicated by the nucleotides in bold.
